# Uterine Leiomyosarcoma Incidentally Diagnosed After Sigmoid Colon Perforation: A Case Report and Review of the Literature Highlighting Individualized Surgical and Oncologic Decision-Making

**DOI:** 10.3390/jpm16070392

**Published:** 2026-07-22

**Authors:** Theodora Palyvou, Ioannis Stefanou, Sotirios Kympouris, Theodora Imant, Dionysia Thermou, Stavriella Seferli, Vasiliki Kanellopoulou, Despoina Chatzopoulou, Katrin Spyropoulou, Nikolaos Kardaras, Milena Iotova, Asimina Ntotsika, Maria-Christina Kapoutsi, Iasonas Priftis, Mara Bouga, Christina Bolanou, Georgios Sygkounas, Spyridon Volteas

**Affiliations:** Department of Surgery, Hippokration General Hospital, Vasilissis Sofias 114, 11527 Athens, Greece; johnstefanou77@gmail.com (I.S.); sotiriskympouris@hotmail.com (S.K.); doraimad1991@gmail.com (T.I.); diwni19@hotmail.com (D.T.); seferstav@gmail.com (S.S.); vcanll@hotmail.com (V.K.); despoinachatz@gmail.com (D.C.); katrin9362@gmail.com (K.S.); nickos.kardaras@gmail.com (N.K.); milena.mvy@gmail.com (M.I.); ntotsika.a@gmail.com (A.N.); mariakapoutsi@gmail.com (M.-C.K.); iasonpriftis@gmail.com (I.P.); marabouga@gmail.com (M.B.); xristinamp.1999@gmail.com (C.B.); giorgos.sigounas@gmail.com (G.S.); igna.kratikixeirourgiki@gmail.com (S.V.)

**Keywords:** uterine leiomyosarcoma, acute abdomen, emergency exploratory laparotomy, sigmoid colon perforation, general surgery, emergency surgery, uterine sarcoma, narrative review

## Abstract

**Introduction:** Uterine leiomyosarcoma (uLMS) is a rare, highly aggressive malignancy arising from the smooth muscle tissue of the uterine wall. It typically presents with abnormal vaginal bleeding, pelvic pain, or a pelvic mass. In rare instances, symptoms may result from local invasion or metastasis. We report a case of uLMS initially diagnosed following colonic perforation due to direct tumor invasion, accompanied by a comprehensive narrative review of the literature on the incidental identification of uterine sarcomas during emergency general surgery to further emphasize the diagnostic challenges, treatment approaches, and need for individualized care in these complex cases. **Case Presentation:** A 45-year-old woman presented to the Emergency Department with acute abdominal pain of several hours’ duration, in the absence of other associated symptoms. An emergency exploratory laparotomy was performed, revealing feculent peritonitis secondary to sigmoid colon rupture, resulting from local invasion by a large uterine mass. A total abdominal hysterectomy with bilateral salpingo-oophorectomy was undertaken, followed by en bloc resection of the sigmoid colon, appendectomy and construction of a terminal colostomy. Her postoperative course was uneventful, and she was discharged on postoperative day eight. Histopathological examination of the specimen revealed a high-grade uterine leiomyosarcoma. Following evaluation by a multidisciplinary oncology board, the patient received adjuvant chemotherapy. **Methods and Results:** A narrative review of the literature was performed to identify cases of uterine sarcomas incidentally diagnosed during emergency surgery performed by general surgeons. Including the present case, six cases were identified. Most patients presented with acute abdomen mimicking gastrointestinal pathology, with diagnosis established intraoperatively or postoperatively. Definitive surgical management was achieved during the initial emergency procedure in all cases. **Conclusion:** Although exceptionally rare, uterine sarcoma should be considered in the differential diagnosis of acute abdomen in female patients undergoing emergency surgery. Awareness of this atypical presentation, the appropriate diagnostic approach, real-time intraoperative adaptability, and individualized multidisciplinary management are essential for ensuring appropriate surgical management and optimizing patient care.

## 1. Introduction

Uterine sarcomas are rare, aggressive malignancies arising from the myometrium or the connective tissue elements of the endometrium, accounting for less than 10% of cancers of the uterine corpus [1]. These tumors include several histologic subtypes, such as leiomyosarcoma (LMS), endometrial stromal sarcoma (ESS), undifferentiated uterine sarcoma, and adenosarcoma, and often behave more aggressively with poorer prognosis compared with the far more common endometrioid adenocarcinoma. While uterine leiomyomas affect 70–80% of women during their lifetime, uterine sarcomas are exceedingly rare. Correcting for hysterectomy prevalence, the age-adjusted incidence of uterine sarcoma among women aged 30–79 years is approximately 2.8 per 100,000 person-years [2]. Large reviews suggest that the prevalence of unexpected leiomyosarcoma identified during surgery for presumed symptomatic leiomyomas ranges from fewer than 1 to 13 per 10,000 procedures [3,4].

Most uterine sarcomas occur in patients over age 40, although cases have been reported in women as young as 20 years old, with a mean age at diagnosis of approximately 60 years [5,6]. Epidemiologic studies have also shown racial disparities, with Black women having approximately a twofold higher incidence of leiomyosarcoma compared with White women [7,8]. Known or suspected risk factors include long-term tamoxifen therapy, which is associated with an increased but small absolute risk for uterine sarcoma, prior pelvic radiation (particularly for carcinosarcoma), and certain hereditary syndromes such as hereditary leiomyomatosis and renal cell carcinoma (HLRCC) or hereditary retinoblastoma, although data remain limited [9,10,11,12,13,14,15]. Notably, leiomyomas are generally not considered precursors to leiomyosarcomas, with rare exceptions in atypical or cellular variants.

Clinically, uterine sarcomas are difficult to differentiate from benign leiomyomas. Most diagnoses are made postoperatively following myomectomy or hysterectomy performed for presumed benign fibroids, although preoperative identification is occasionally possible via endometrial sampling. Typical presenting features include abnormal uterine bleeding, pelvic pain or pressure, and uterine mass; less commonly, abdominal distension, constipation, urinary symptoms, or prolapse of the tumor through the cervix may occur [1,16,17]. Rarely, patients are asymptomatic. In advanced cases, manifestations may result from local invasion or distant metastasis, which are not seen in benign leiomyomas. Data from a large series of over 1000 uterine sarcoma cases indicate that approximately 60% present at stage I, 16% at stage II–III, and 22% at stage IV [1].

Although uterine sarcomas have been extensively described in elective gynecologic settings, a rare subset of uterine sarcomas presents as an acute abdominal emergency requiring exploratory laparotomy performed by general surgeons. These cases often simulate severe gastrointestinal conditions, including bowel perforation, hemoperitoneum, or diffuse peritonitis. In such scenarios, the diagnosis of a primary uterine sarcoma is frequently unsuspected prior to surgery and is typically identified postoperatively through histopathological analysis, posing diagnostic and management challenges. Given the exceptional rarity of these presentations and the limited published evidence, a systematic consolidation of reported cases is crucial for improving recognition, supporting operative planning, and providing guidance for multidisciplinary management strategies.

In emergency surgical settings, acute abdomen is commonly approached through standardized diagnostic and therapeutic algorithms primarily focused on gastrointestinal pathology. However, rare malignancies such as uterine sarcomas may present with highly atypical clinical and intraoperative findings that fall outside conventional diagnostic pathways. In such scenarios, rigid “one-size-fits-all” approaches may be insufficient, emphasizing the importance of individualized clinical assessment, real-time intraoperative adaptability, and patient-specific multidisciplinary decision-making. These challenging presentations highlight the growing role of personalized surgical care in emergency settings, where management strategies may need to be tailored according to intraoperative findings, extent of disease, and patient condition.

The unusual emergency presentation in our case highlighted this diagnostic gap and prompted a narrative review of the literature, focusing on cases of unexpected primary uterine sarcomas diagnosed during emergency exploratory laparotomy performed by general surgeons. The aim of this review was to provide a comprehensive summary of clinical presentations, intraoperative findings, histologic subtypes and patient outcomes, thereby reinforcing the diagnostic challenges of these cases and the importance of maintaining heightened clinical awareness in acute surgical settings.

## 2. Methods—Literature Search Strategy

A narrative review of the literature was conducted to identify published cases of primary uterine sarcomas that were incidentally diagnosed during emergency exploratory laparotomy performed for general surgical indications. The aim of this review was to summarize clinical presentations, intraoperative findings, surgical management strategies, and postoperative outcomes in cases where uterine sarcoma was not suspected preoperatively.

A comprehensive literature search was performed using the electronic databases PubMed/MEDLINE, Scopus, and Web of Science, covering the period from January 1990 to May 2026; the search was executed in May 2026. No language restrictions were applied. The search strategy combined free-text terms and Medical Subject Headings (MeSH), including *“uterine sarcoma”*, *“uterine leiomyosarcoma”*, *“uterine carcinosarcoma”*, *“acute abdomen”*, *“emergency surgery”*, *“exploratory laparotomy”*, *“bowel perforation”*, *“hemoperitoneum”*, *“uterine rupture”*, and *“incidental diagnosis”*. Reference lists of relevant publications were manually screened to identify additional eligible articles.

***Inclusion criteria**:* Studies were included if they reported cases of primary uterine sarcoma, involved emergency exploratory laparotomy for non-gynecologic indications, and provided intraoperative or postoperative histopathologic confirmation of uterine sarcoma.

***Exclusion criteria***: Studies were excluded if they did not involve exploratory laparotomy (n = 3), had a preoperative diagnosis of uterine sarcoma (n = 2), were limited to obstetric settings (n = 1), involved elective gynecologic surgery (n = 9), reported metastatic or non-uterine sarcomas (n = 7), or were reviews, editorials, or conference abstracts without sufficient clinical details (n = 3).

Data were extracted from each included report regarding patient age, presenting symptoms, indication for emergency surgery, intraoperative findings, type of surgical procedure performed, histologic subtype, and postoperative outcomes or follow-up when available. Due to heterogeneity in case presentations and reporting, quantitative synthesis was not performed. Findings were summarized descriptively and synthesized narratively to highlight patterns of presentation, intraoperative decision-making, and outcomes.

**Figure 1:**
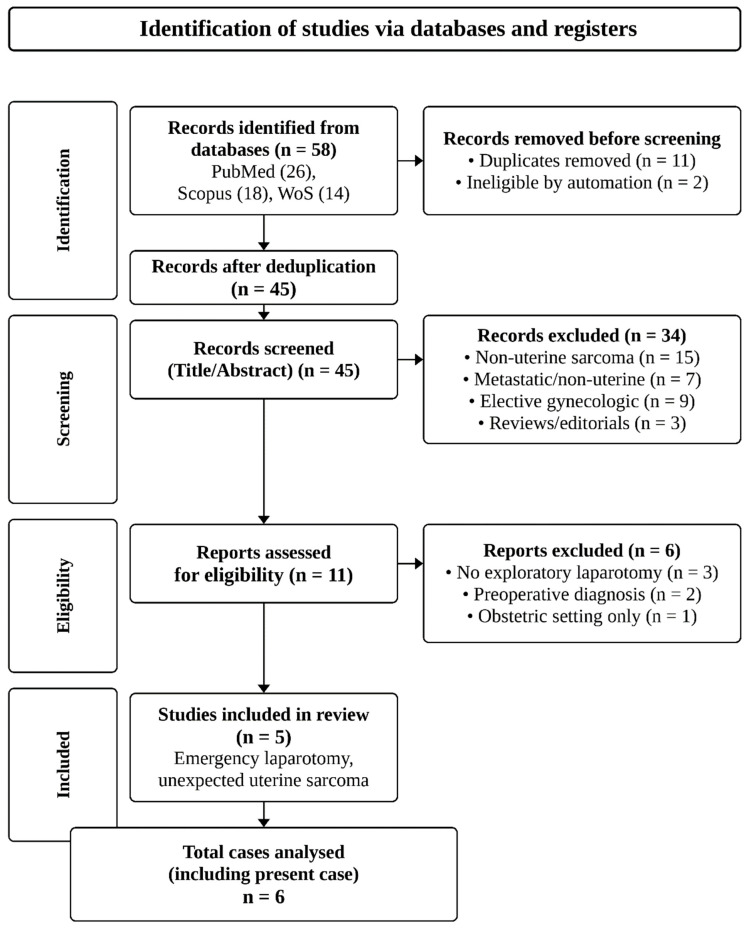
Flowchart of the literature search and study selection process for unexpected primary uterine sarcomas diagnosed during emergency exploratory laparotomy.

The literature search and study selection process are summarized in Figure 1. The characteristics of the identified cases, along with the present case, are detailed in the Results section and summarized in Table 1. This approach provides a comprehensive overview of the rare but clinically significant scenario of unexpected intraoperative diagnosis of primary uterine sarcomas in emergency general surgery settings.

## 3. Case Presentation

A 45-yearold woman presented to the Emergency Department with sudden-onset acute abdominal pain of several hours’ duration, in the absence of other associated symptoms. Her medical history was unremarkable. Physical examination revealed diffuse abdominal tenderness on both superficial and deep palpation with signs consistent with peritoneal irritation, including rebound tenderness. Laboratory evaluation showed leukocytosis with neutrophilia and elevated inflammatory markers. Subsequent contrast-enhanced abdominal computed tomography (CT) demonstrated findings suggestive of hollow viscus perforation, most likely involving the sigmoid colon, along with marked perisigmoid fat stranding and associated intra-abdominal fluid collections.

**Figure 2:**
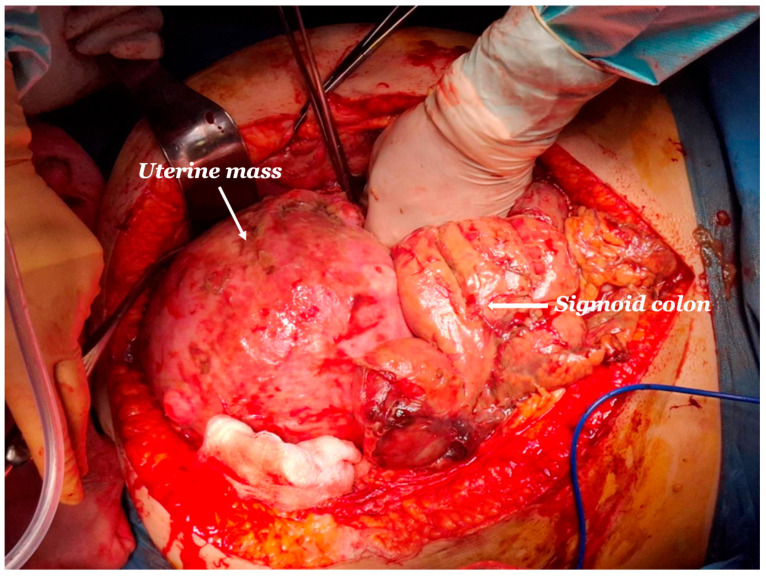
Intraoperative image demonstrating a large uterine mass identified during emergency exploratory laparotomy.

An emergency exploratory laparotomy was performed, revealing feculent peritonitis secondary to sigmoid colon rupture occurring in the context of deep tumor infiltration of the sigmoid colonic wall, with histopathological extension reaching the muscularis propria by a large uterine mass, resulting in mechanical compromise of colonic wall integrity (Figure 2). Surgical management included a total abdominal hysterectomy with bilateral salpingo-oophorectomy, en bloc resection of the affected sigmoid colon, appendectomy due to dense adhesions to inflamed surrounding tissues and construction of a terminal colostomy. Immediately postoperatively, the patient was transferred to the intensive care unit (ICU), where she remained intubated for 48 h. Following successful weaning from mechanical ventilation, she was extubated and returned to the surgical ward. Her postoperative course was uneventful, with early mobilization and prompt resumption of oral intake. She was discharged in good clinical condition on postoperative day eight.

**Figure 3:**
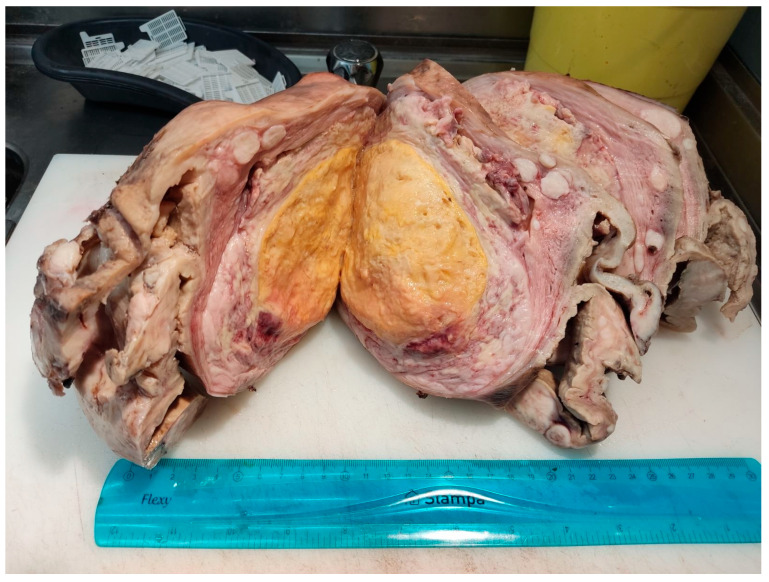
Gross pathological specimen of the resected uterine mass, sectioned in the pathology laboratory for histopathological evaluation.

Histopathological examination of the specimen (Figure 3) revealed a high-grade uterine leiomyosarcoma with a maximum diameter of 22 cm, characterized by an increased mitotic index (>14 MF/10 HPF), extensive necrosis, and hemorrhagic infiltration. The tumor also demonstrated extension to the left ovary, left fallopian tube, and the muscularis propria of the sigmoid colon. The right adnexa was free of neoplastic infiltration, while the appendix showed a focal area of endometriosis. Immunohistochemical analysis revealed the following profile: Desmin (+), SMA (focally +), caldesmon(+), CD10(+), CD117(−), DOG-1 (−), Cyclin D1 (−), Ki-67 67–70%.

Following evaluation by a multidisciplinary oncology board, the patient received adjuvant chemotherapy. At subsequent follow-up within the year, she remained disease-free with satisfactory colostomy function.

This case represents an exceptionally rare presentation of uterine sarcoma as bowel perforation, highlighting the diagnostic challenges encountered when a primary uterine tumor is incidentally discovered intraoperatively during general surgery procedures. This case prompted a comparison with other similar reports in the literature, focusing on unexpected uterine sarcomas identified incidentally during emergency general surgical interventions.

## 4. Results

A total of six cases of unexpected primary uterine sarcoma diagnosed during emergency exploratory laparotomy were identified in the literature, including the present case. Patient age ranged from 43 to 69 years. Acute abdominal pain was the most common presenting symptom and was frequently accompanied by signs of peritoneal irritation, hemoperitoneum, pneumoperitoneum, or hemodynamic instability. In most cases, preoperative clinical evaluation and imaging suggested gastrointestinal pathology, such as hollow-viscus perforation or intra-abdominal hemorrhage, while a gynecologic malignancy was rarely suspected prior to surgery. The key characteristics of the identified cases, including the present case, are summarized in Table 1.

Indications for emergency laparotomy included acute abdomen of unclear origin, suspected bowel perforation, and unexplained intra-abdominal bleeding. Intraoperative findings demonstrated heterogeneous disease patterns. Several cases involved spontaneous uterine rupture with extensive tumor necrosis and friable uterine tissue, whereas others revealed direct invasion of adjacent organs, most commonly the sigmoid colon. Purulent or hemorrhagic peritoneal fluid was frequently encountered, reinforcing the initial suspicion of gastrointestinal pathology.

Surgical management consistently included total abdominal hysterectomy, with bilateral salpingo-oophorectomy performed in the majority of patients. In cases with local organ invasion, en bloc resection was required to achieve adequate surgical control, including segmental bowel resection when indicated. Despite the unexpected intraoperative diagnosis, definitive surgical treatment was completed during the initial emergency procedure in all reported cases.

Histopathological examination confirmed high-grade uterine sarcoma in all patients. Leiomyosarcoma was the predominant histologic subtype, although undifferentiated uterine sarcoma and uterine carcinosarcoma were also identified. Tumors were characterized by high mitotic activity, extensive necrosis, and hemorrhagic infiltration. Immunohistochemical analysis, when available, supported smooth muscle differentiation with markers such as desmin, smooth muscle actin, and caldesmon, accompanied by high proliferative indices.

Postoperative recovery was generally uneventful. Most patients received adjuvant chemotherapy following multidisciplinary oncologic evaluation. Short-term follow-up, when reported, was favorable in several cases, although oncologic outcomes varied due to limited follow-up duration.

## 5. Discussion

The present review highlights an exceptionally rare but clinically important scenario in which primary uterine sarcomas present as acute abdominal emergencies requiring exploratory laparotomy, most often performed for suspected gastrointestinal pathology [1,6,7]. Across the identified cases, including the present report, the preoperative diagnosis was consistently misleading, with imaging and clinical findings suggesting hollow-viscus perforation, intra-abdominal hemorrhage, or generalized peritonitis rather than a gynecologic malignancy. This diagnostic discordance underscores the ability of aggressive uterine sarcomas to mimic common surgical emergencies, thereby masking their true origin until intraoperative exploration or postoperative histopathologic evaluation.

Despite heterogeneity in tumor subtype and intraoperative findings, several unifying patterns emerged. Acute abdominal pain predominated as the presenting feature, while intraoperative assessment frequently revealed necrotic, friable uterine masses, spontaneous uterine rupture, or direct invasion of adjacent organs. These findings emphasize the aggressive biological behavior of uterine sarcomas and their potential to present outside traditional gynecologic pathways. Importantly, definitive surgical management was achieved during the initial emergency procedure in all reported cases, underscoring the critical role of timely surgical decision-making in unstable patients or in settings of diagnostic uncertainty. Collectively, these observations support the recognition of uterine sarcoma as a rare but relevant differential diagnosis in acute abdomen and form the basis for discussing diagnostic challenges, intraoperative management strategies, and broader clinical implications in emergency surgical practice.

Primary uterine sarcomas rarely present through emergency surgical pathways, yet when they do, their clinical manifestation can be indistinguishable from common gastrointestinal emergencies. In the cases included in this review, preoperative symptoms and laboratory findings were nonspecific and frequently consistent with acute abdomen, sepsis, or intra-abdominal bleeding. Cross-sectional imaging, although essential in emergency assessment, predominantly suggested gastrointestinal pathology, including bowel perforation or unexplained hemoperitoneum, with limited indication of a gynecologic source. These observations illustrate the limitations of preoperative diagnostics in identifying uterine sarcomas under emergent conditions.

The diagnostic challenge is further compounded by the rarity of uterine sarcomas and the absence of gynecologic symptoms in several cases, particularly in postmenopausal patients or those with a known history of uterine leiomyomas. As a result, initial management is often directed toward presumed gastrointestinal disease, and surgical exploration is undertaken by general surgeons without preoperative suspicion of a uterine malignancy. This delay in recognition does not reflect diagnostic error but rather the atypical clinical behavior of these tumors, which can rupture, necrose, or invade adjacent organs, thereby producing misleading radiologic and intraoperative findings. Awareness of this diagnostic pitfall is therefore essential, as early intraoperative recognition may influence surgical strategy, extent of resection, and timely involvement of gynecologic oncology services [18,19,20].

Emergency laparotomy in the context of acute abdomen requires rapid decision-making under conditions of diagnostic uncertainty. In the reported cases, definitive surgical management was initiated at the time of the initial operation, despite the unexpected identification of a uterine malignancy. Total abdominal hysterectomy, with or without bilateral salpingo-oophorectomy, constituted the cornerstone of surgical treatment. When adjacent organ invasion or gross contamination was identified, en bloc resection was required to achieve adequate source control and oncologic clearance, as in our present case. Surgical management was individualized according to intraoperative findings, extent of local invasion, degree of contamination, and overall patient condition rather than predetermined operative protocols.

These findings highlight the critical role of the operating surgeon in adapting intraoperative strategy when encountering unexpected gynecologic pathology. Although uterine sarcomas fall outside the routine scope of emergency general surgery, failure to recognize their malignant potential may result in incomplete resection or delayed definitive treatment. Multidisciplinary collaboration, particularly intraoperative consultation with gynecologic specialists when available, can facilitate appropriate surgical decision-making. Importantly, the successful completion of definitive surgical treatment during the initial emergency procedure in all reported cases demonstrates the feasibility of prompt, decisive intervention even in unstable or complex scenarios. This emphasizes the need for general surgeons to maintain situational awareness and flexibility when managing atypical sources of acute abdomen.

Beyond their atypical clinical presentation, the cases included in this review underscore the aggressive biological behavior of uterine sarcomas. Most tumors were high-grade, with leiomyosarcoma representing the predominant histologic subtype, while undifferentiated uterine sarcoma and uterine carcinosarcoma were also observed. Common histopathologic features included high mitotic activity, extensive necrosis, and hemorrhagic infiltration, findings that correlate with rapid tumor progression and poor prognosis. In several cases, direct invasion of adjacent organs or anatomical structures was identified at the time of emergency surgery, reflecting advanced local disease despite the absence of a preoperative oncologic diagnosis [6,7].

In the present case, the markedly elevated Ki-67 proliferation index (67–70%) further supported the aggressive biological behavior of the tumor. High Ki-67 expression in uterine leiomyosarcoma has been associated with increased proliferative activity, higher recurrence risk, and poorer oncologic outcomes. Such histopathological characteristics may influence individualized postoperative management, including decisions regarding adjuvant chemotherapy and surveillance strategies. Emerging molecular and genomic profiling approaches may further contribute to personalized risk stratification and tailored therapeutic planning in uterine sarcomas.

These histologic characteristics have important implications for postoperative management. The aggressive nature of uterine sarcomas necessitates prompt referral for multidisciplinary oncologic evaluation, even when definitive resection has been achieved during the emergency procedure. Adjuvant chemotherapy was administered in most reported cases, reflecting the need for coordinated postoperative care. Although long-term outcomes could not be uniformly assessed due to limited follow-up, the combination of high-grade histology and advanced local invasion reinforces the importance of early surgical control and timely oncologic intervention once the diagnosis is established.

The findings of this review carry important clinical implications for general surgeons, who are often the first to encounter these patients in emergency surgical settings. Although uterine sarcomas are rare, they should be considered in the differential diagnosis of acute abdomen in female patients, particularly when intraoperative findings reveal necrotic uterine tissue, spontaneous uterine rupture, or unexplained pelvic involvement. Early recognition of a potential gynecologic origin may influence intraoperative decision-making, including the extent of resection and the need for gynecologic consultation.

From a personalized medicine perspective, these cases highlight the necessity of tailoring surgical management to intraoperative findings rather than relying solely on preoperative assumptions. Emergency surgical care in such scenarios should remain adaptable and individualized, balancing immediate source control with oncologic principles according to patient-specific intraoperative findings. Heightened awareness of this rare presentation may facilitate earlier diagnosis, appropriate surgical intervention, and timely multidisciplinary management, ultimately improving patient outcomes in an otherwise challenging and unpredictable clinical context. Whereas previously reported cases predominantly presented with tumor rupture or hemoperitoneum, the present case was characterized by sigmoid colon perforation secondary to direct tumor invasion.

A critical cross-case analysis of the six identified cases reveals several important patterns that extend beyond a simple case enumeration. With respect to the mechanism of acute presentation, three cases involved spontaneous uterine rupture with hemoperitoneum [18,19,22], one involved rupture with purulent peritonitis [20], one involved fundal uterine perforation with pneumoperitoneum [21], and the present case uniquely demonstrated direct tumor infiltration of the sigmoid colon wall leading to feculent peritonitis—a mechanistically distinct and previously unreported presentation. Regarding histologic subtype, leiomyosarcoma predominated (four of six cases), with one case of undifferentiated uterine sarcoma and one carcinosarcoma, reflecting the heterogeneous spectrum of uterine sarcomas capable of producing emergency presentations. The extent of surgical resection varied accordingly: total abdominal hysterectomy with or without bilateral salpingo-oophorectomy was performed in all cases, while en bloc multivisceral resection was required exclusively in the present case due to sigmoid colon involvement, underscoring the importance of individualized intraoperative decision-making. Short-term oncologic outcomes, where reported, were variable: one patient experienced disease progression during follow-up, one was disease-free at six months, and the present patient remained disease-free at one-year follow-up following adjuvant chemotherapy. These differences likely reflect tumor biology, stage at presentation, and adequacy of resection rather than any systematic pattern, and reinforce the need for individualized postoperative oncologic management in each case.

An important consideration in cases of uterine sarcoma presenting with perforation or rupture is the risk of peritoneal dissemination. Intraoperative spillage of tumor contents, uterine manipulation, and peritoneal contamination by necrotic or hemorrhagic tumor tissue may theoretically facilitate peritoneal seeding and adversely influence oncologic outcomes. Although evidence specifically addressing the impact of intraoperative spillage on survival in emergency settings remains limited, data from elective surgery suggest that morcellation and tumor spillage are associated with upstaging and worse prognosis in uterine leiomyosarcoma [23,24]. In the emergency context, complete prevention of spillage may not always be feasible; however, meticulous peritoneal lavage, avoidance of unnecessary tumor fragmentation, and prompt postoperative multidisciplinary oncologic evaluation are recommended. These considerations further reinforce the importance of early intraoperative recognition of a potential gynecologic malignancy and the involvement of gynecologic oncology services whenever possible.

## 6. Limitations

This review has several limitations that should be acknowledged. First, the analysis is based on a limited number of published cases, reflecting the rarity of unexpected uterine sarcoma presenting as an acute surgical emergency. As a result, the findings may not be generalizable and should be interpreted with caution. Second, the included reports are heterogeneous with regard to clinical presentation, surgical management, histopathological assessment, and follow-up duration, which limits meaningful comparison across cases. In addition, follow-up data were incomplete or short-term in several reports, precluding reliable assessment of long-term oncologic outcomes. Finally, as this is a narrative review incorporating a single institutional case, the possibility of publication and reporting bias cannot be excluded. Formal FIGO staging was also not reported in the majority of the included cases, limiting comparative oncologic analysis across the identified cases. The marked heterogeneity among the reported cases further supports the need for individualized management strategies rather than standardized treatment algorithms in these rare and unpredictable emergency presentations. Nevertheless, the synthesis of available evidence provides valuable insights into a rare and clinically challenging presentation that may inform surgical awareness and decision-making in emergency settings.

## 7. Conclusions

Unexpected uterine sarcoma presenting as an acute abdominal emergency represents a rare but clinically significant diagnostic challenge. The findings of this review demonstrate that these tumors may mimic common gastrointestinal emergencies, often leading to intraoperative diagnosis during emergency exploratory laparotomy performed by general surgeons. In such scenarios, real-time clinical personalization, adaptive surgical strategy, and case-based multidisciplinary decision-making are essential to achieve appropriate source control and oncologic management. Awareness of these atypical presentations may facilitate earlier recognition and timely individualized care, ultimately improving outcomes in patients with this aggressive and unpredictable malignancy.

## Figures and Tables

**Table 1:** Reported cases of unexpected diagnosis of primary uterine sarcoma following emergency exploratory laparotomy.

Author (Year)	Age (Years)	Presenting Symptoms	Indication for Exploratory Laparotomy	Intraoperative Findings	Surgical Procedure Performed	Histopathological Diagnosis	Outcome/Follow-Up
Hicks et al., 2010 [18]	52	Acute abdomen, anemia	Hemoperitoneum	Large necrotic uterine mass	Total abdominal hysterectomy (TAH)	Undifferentiated uterine sarcoma	Limited follow-up reported
Takeuchi et al., 2014 [19]	59	Sudden abdominal pain, shock	Acute abdomen of unclear origin	Uterine perforation with active intraperitoneal bleeding	TAH	Uterine leiomyosarcoma	Disease progression during follow-up
Boussouni et al., 2016 [20]	43	Acute abdominal pain, peritonitis	Suspected hollow-viscus perforation	Spontaneous uterine rupture with generalized peritonitis	TAH + Bilateral Salpingo-Oophorectomy (BSO)	Uterine leiomyosarcoma	Uneventful recovery; adjuvant therapy administered
Sassaman et al., 2024 [21]	69	Acute abdominal pain, sepsis, pneumoperitoneum (no gynecologic symptoms)	Suspected perforated viscus with sepsis	Enlarged necrotic uterus with fundal perforations and purulent peritoneal fluid	TAH + BSO + partial omentectomy	Uterine carcinosarcoma (FIGO stage IIIA) with heterologous rhabdomyosarcoma component	Discharged on postoperative day 13; referred for adjuvant chemo/radiotherapy planned
Hasanzadeh et al., 2025 [22]	44	Acute abdominal pain, hemoperitoneum	Suspected intra-abdominal hemorrhage	Ruptured intramural/subserosal uterine mass	TAH + BSO	Uterine leiomyosarcoma	Disease-free at 6 month follow-up
Present case, 2024	45	Acute abdominal pain	Feculent peritonitis from suspected colonic perforation	Direct sigmoid colon invasion by large uterine mass	TAH + BSO + en bloc sigmoidectomy + colostomy + appendectomy	High-grade uterine leiomyosarcoma	Adjuvant chemotherapy initiated; disease-free at 1-year follow-up

## Data Availability

No new data were created or analyzed in this study. Data sharing is not applicable to this article.
